# Upregulation of *SMYD3* and *SMYD3* VNTR 3/3 polymorphism increase the risk of hepatocellular carcinoma

**DOI:** 10.1038/s41598-020-59667-z

**Published:** 2020-02-18

**Authors:** Mai Thanh Binh, Nghiem Xuan Hoan, Dao Phuong Giang, Hoang Van Tong, C.-Thomas Bock, Heiner Wedemeyer, Nguyen Linh Toan, Mai Hong Bang, Peter G. Kremsner, Christian G. Meyer, Le Huu Song, Thirumalaisamy P. Velavan

**Affiliations:** 1 Institute of Tropical Medicine, University of Tübingen, Tübingen, Germany; 2 Vietnamese-German Center for Medical Research (VGCARE), Hanoi, Vietnam; 3 108 Military Central Hospital, Hanoi, Vietnam; 4 Vietnam Military Medical University, Hanoi, Vietnam; 5 Department of Infectious Diseases, Robert Koch Institute, Berlin, Germany; 6 Department of Gastroenterology and Hepatology, Essen University Hospital, University of Duisburg-Essen, Essen, Germany; 7 Duy Tan University, Da Nang, Vietnam

**Keywords:** Hepatocellular carcinoma, Hepatitis B

## Abstract

SMYD3 (SET and MYND domain-containing protein 3) is involved in histone modification, which initiates oncogenesis by activating transcription of multiple downstream genes. To investigate associations of variable numbers of tandem repeats (VNTR) variants in the *SMYD3* gene promoter, SMYD3 serum levels and *SMYD3* mRNA expression in hepatitis B virus (HBV) infection and clinical progression of related liver disease. *SMYD3* VNTRs were genotyped in 756 HBV patients and 297 healthy controls. SMYD3 serum levels were measured in 293 patients and *SMYD3* mRNA expression was quantified in 48 pairs of hepatocellular tumor and adjacent non-tumor liver tissues. Genotype *SYMD3* VNTR 3/3 was more frequent among HCC patients than in controls (*P*_adjusted_ = 0.037). SMYD3 serum levels increased according to clinical progression of liver diseases (*P* = 0.01); HCC patients had higher levels than non-HCC patients (*P* = 0.04). Among patients with *SMYD3* VNTR 3/3, HCC patients had higher SMYD3 levels than others (*P* < 0.05). *SMYD3* mRNA expression was up-regulated in HCC tumor tissues compared to other tissues (*P* = 0.008). In conclusion, upregulation of *SMYD3* correlates with the occurrence of HCC and *SMYD3* VNTR 3/3 appears to increase the risk of HCC through increasing SMYD3 levels. SMYD3 may be an indicator for HCC development in HBV patients.

## Introduction

Although effective hepatitis B virus (HBV) vaccines are in use worldwide, HBV-related liver diseases are still a major health concern with approximately 257 million chronic infections and 887,000 deaths in 2015^1^. HBV is the main cause of primary hepatocellular carcinoma (HCC)^2^. The risk of HCC development is approximately 40 times higher in chronic HBV carriers (CHB) than in non-carriers^3^. Vietnam has a high prevalence of HBV infections, ranging from 10–20% in the general population and 20–40% among high-risk groups^2,4^. As a result, Vietnam is one of the countries with a high incidence of HCC with >25,000 new cases reported in 2018^5,6^.

Further to viral factors contributing to cancer development, methylation of histone proteins is an important mechanism involved in multiple types of cancers including HCC^7,8^. Methylation of histone proteins at lysine residues can lead to chromatin remodelling, transcription, and signal transduction^9^. The SET and MYND domain containing proteins (SMYDs) belong to a family of the multi-domain SET-containing histone lysine methyltransferases and play a crucial role in histone methylation. To date, five SMYD family members have been recognized (SMYD1-SMYD5). SMYD3 is the most important member, as several findings have demonstrated its role in tumor cell growth and its increased expression in various cancers.

SYMD3 promotes dimethylation and trimethylation of histone H3 lysine 4 (H3K4), which initiates oncogenesis by activating transcription of multiple downstream target genes^10,11^. SYMD3 overexpression causes cell proliferation, migration and adhesion, whereas suppression by RNAi inhibits cell proliferation and migration, indicating that SMYD3 plays an important role in carcinogenesis^12–14^. SYMD3 was found upregulated in HCC, colorectal and in breast and lung cancers^12,15,16^. *In vitro* interaction of SMYD3 with HBV has been demonstrated, showing that SMYD3 expression was upregulated by hepatitis B x protein (HBx) in HepG2 cells, promoting HCC development and clinical progression^17^. Moreover, SMYD3 is a HBx-interacting protein, and this interaction induces activation of the activator protein 1 (AP-1), which increases the risk of HCC formation^18,19^.

The common variable number of tandem repeat (VNTR) CCGCC sequence in the *SMYD3* promoter region is the binding site for the E2F transcription factor 1 (E2F-1) and shown to be a susceptibility factor for human malignancies^20^. It has been suggested that, compared to the genotype containing two copies, the genotype involving three copies of the CCGCC motif might enhance the binding affinity to E2F-1 and, as a result, promote cancer progression by activating transcription of multiple oncogenes such as myc gene (myc), signal transducer and activator of transcription 3 (STAT3) and β-catenin (β-cat)^14,20,21^. Moreover, the *VNTR* genotype *3/3* was associated with a higher risk of colorectal cancer, HCC, breast cancer, ovarian cancer and esophageal squamous cell carcinoma^20,22,23^. Particularly in liver diseases, *VNTR* genotype *3/3* contributed an over 3-fold increased risk of HCC in a Japanese and a Chinese population^20,24^. The present study aimed to investigate the association of VNTR polymorphisms in the *SMYD3* promoter and SMYD expression with HBV infection and clinical progression of HBV-related liver diseases, in particular progression to HCC.

## Patients and Methods

### Patients

Seven hundred and fifty-six Vietnamese chronic HBV-infected patients enrolled between 2013 and 2015 at the 108 Military Central Hospital, Hanoi, Vietnam, were recruited. All patients were negative for antibodies for hepatitis C virus (anti-HCV) and antibodies for human immunodeficiency virus (anti-HIV) nor had a history of alcohol or drug abuse. Patients were categorized into the subgroups of chronic hepatitis B (CHB) without liver cirrhosis (LC) or HCC (n = 246), HBV-related LC patients (n = 174) and HBV-related HCC patients (n = 336). The clinical and diagnostic characteristics of the study group have been described previously^25^. LC and HCC patients were further classified according to Child-Pugh scores A, B, and C^26^ and HCC patients were categorized according to the Barcelona Clinic Liver Cancer (BCLC) staging system^27^. The control group (healthy controls; HC) consisted of 297 healthy blood donors. All HCs were HBV surface antigen (HBsAg), anti-HCV and anti-HIV negative. Five mls of venous blood were collected from each participant. Serum was stored at −80 °C until further use.

In order to determine *SMYD3* mRNA expression, pairs of HCC tumor and adjacent non-tumor tissue specimens were obtained from 48 HCC patients who underwent surgery at the 108 Military Central Hospital. The tumor stage was scored following the BCLC system^28^.

### Ethics statement

Informed written consent was obtained from all participants after detailed explanation of the study at the time of blood sampling. The study protocol conforms to the ethical guidelines of the 1975 Declaration of Helsinki as reflected in a priori approval by the institution’s human research committee. The Institutional Review Board of the 108 Military Central Hospital, Hanoi, Vietnam (108MCH/Res/Epi-HBV-HDV-HEV-D2-14-03-2014) and the Ethics committee of University of Tübingen (206/2012B02) approved the study.

### *SMYD3* VNTR genotyping

Genomic DNA of all participants was isolated from whole blood (DNA purification kit; Qiagen, Hilden, Germany). The promoter region of the *SMYD3* gene was amplified using primers SMYD3_F (5′-CGC CTG TCT TCT GCG CAG TCG-3′) and SMYD3_R (5′-CCC GAG AAG GCA GCG GTC G-3′). Amplicons underwent DNA sequencing.

### Quantification of SMYD3 serum levels by ELISA

Of the 756 patients, 293 individuals were tested for SMYD3 serum levels, measured by a commercially available human SMYD3 sandwich ELISA kit (Wuhan Fine Biological Technology Co. Ltd, Wuhan City, China). In order to determine SMYD3 concentrations, a standard curve was plotted (https://www.curveexpert.net/) based on mean of OD values and the known concentration of the standards. Finally, SMYD3 concentrations were interpolated based on the standard curve. The detectable range of the kit was 31.25–2000 pg/ml.

### Quantification of *SMYD3* mRNA by RT-PCR

Total RNA of the liver tissues was extracted with Trizol reagent (Life Technologies, Carlsbad, CA, USA), followed by reverse transcription into cDNA (QuantiTect Reverse Transcription Kit; Qiagen, Hilden, Germany). *SMYD3* mRNA levels were assessed through quantification of *SMYD3* cDNA by qRT-PCR using SYBR Green PCR master mix (Bioline, Luckenwalde, Germany). The *GAPDH* gene (glyceraldehyde-3-phosphate dehydrogenase) was used as reference. Primer sequences were SMYD3_F: 5′-GTT GGC CTA TAT CCC AGT ATC TCT TTG CTC -3′, and SMYD3_R: 5′-ACC AGT TAG CAT ATC AGC ATC CTT GTC CTG -3′, GAPDH_F: 5′-CCA CCC ATG GCA AAT TCC ATG GCA-3′ and GAPDH_R: 5′-TCT AGA CGG CAG GTC AGG TCC ACC-3′. All qRT-PCR reactions were performed in duplicate and repeated twice (LightCycler® 480 real-time PCR system; Roche, Basel, Switzerland). The fold change of *SMYD3* mRNA was normalized based upon the ΔΔCt method against expression of Glyceraldehyde 3-phosphate dehydrogenase (*GAPDH)*.

### Statistical analysis

Chi-square and Fisher´s exact tests were used to test for differences of categorical variables. Kruskal-Wallis and Mann-Whitney-Wilcoxon tests were applied to compare quantitative variables. Hardy-Weinberg equilibrium was assessed. Binary logistic regression models adjusted for age and gender were applied to determine *SMYD3* VNTR associations with HBV-related liver diseases. Adjusted odds ratios (OR) with 95% confidence intervals (CI) were calculated. Paired-samples t-test was used to compare *SMYD3* mRNA levels in tumor and adjacent non-tumor tissues. A linear regression model was applied to analyze the relationship of clinical parameters of patients and SMYD3 levels. Pearson’s correlation coefficient test was used to analyze correlations between SMYD3 levels and clinical parameters. Statistical analyses were performed using SPSS version 22 (SPSS Statistics, IBM, Armonk, NY, USA) and GraphPad Prism 7 (http://www.graphpad.com). Significance was set at *P* < 0.05.

## Results

### Patient characteristics

Demographic, laboratory and clinical parameters of the 1053 study participants are summarized in Table 1. In the HC group, the mean age was 43 years (range: 18–69), and majority of HCs were male (66.7%). Of the 756 patients, 630 (83.3%) were male; the mean age was 52 years (12–91). The median age of patients and the proportion of males increased according to the degree of progression of liver diseases (*P* < 0.0001). As expected, albumin, prothrombin levels and platelet counts were higher among CHB patients compared to the other patient subgroups (*P* < 0.0001). HBV DNA levels did not differ among patient subgroups (*P* = 0.4). Higher total bilirubin and direct bilirubin were observed in LC patients compared to the other groups (*P* < 0.0001). Alpha-fetoprotein (AFP) levels were significantly higher in HCC patients compared to CHB and LC patients (*P* < 0.0001) (Table 1).

**Table 1:** Demographic and clinical characteristics of healthy controls and HBV patients.

Characteristics	HC (n = 297)	HBV patients (n = 756)	CHB (n = 246)	LC (n = 174)	HCC (n = 336)	*P* value
Age (years)	43 [16–69]	52 [12–91]	41 [12–85]	56 [20–86]	57 [15–91]	<0.0001^#^
Male (%)	66.7	83.3	75.2	82.8	93.7	<0.0001^β^
Child-Pugh	NA					
Child A			NA	53/169	249/335	
Child B			NA	75/169	65/300	
Child C			NA	41/169	21/335	
Missing			NA	5	1	
**Clinical parameters**						
AST (IU/L)	NR	132 [14–6206]	187 [14–6206]	119 [15–1221]	101 [17–983]	<0.0001^#^
ALT (IU/L)	NR	132 [8–3390]	222 [9–3390]	82 [8–1426]	72 [11–1095]	0.04^#^
Total bilirubin (µmol/L)	NR	39.1 [4.1–571]	34 [5.5–551]	65.2 [4.1–571]	29.4 [4.3–392]	<0.0001^#^
Direct bilirubin (µmol/L)	NR	17.2 [0.4–349]	16.1 [0.7–349]	29.5 [0.4–291]	11.6 [0.4–247.3]	<0.0001^#^
Albumin (g/L)	NR	37 [15–48]	42 [25–48]	31.8 [15–47]	37 [15–48]	<0.0001^#^
Prothrombin (% of standard)	NR	82 [13–269]	94 [17–267]	60 [13–101]	84 [20–269]	<0.0001^#^
						<0.0001^#^
WBC (×10^3^/mL)	NR	**6.6 [1.7–20.5]**	**6.7 [4.1–13.44]**	**6.2 [1.7–20.5]**	**6.8 [2.7–17.9]**	<0.0001^#^
RBC(×10^6^/mL)	NR	**4.5 [1.7–6.8]**	**4.9 [3.1–6.8]**	**3.9 [1.9–6.7]**	**4.5 [1.7–6.8]**	<0.0001^#^
PLT (×10^3^/ml)	NR	174 [17–441]	218 [66–379]	106 [17–441]	177 [34–432]	<0.0001^#^
HBV DNA (log_10_ copies/ml)	NR	5.1 [1–10]	5.2 [2–10]	5 [1–10]	5.1 [1–9]	0.4^#^
AFP (IU/L)	NR	142 [1–4029]	7.4 [1–250]	40 [1.18–707]	280 [1–4029]	<0.0001^#^

CHB, chronic hepatitis B; LC, liver cirrhosis; HCC, hepatocellular carcinoma; HC, healthy control; RBC, red blood cells; WBC, white blood cells; PLT, platelets. AST and ALT, aspartate and alanine aminotransferase; AFP, alpha-fetoprotein; NR, normal range, NA, not applicable. Values given are medians and ranges. (#) Kruskal-Wallis test (β): chi-square test.

### *SMYD3* VNTRs and HBV-related liver diseases

The allele frequencies and genotype distributions of *SMYD3* VNTR polymorphisms are given in Table 2. Allele frequencies of *VNTR 2* and *3* were 21.7% and 78.3% among controls and 12% and 88% in HBV patients, respectively. The three tandem repeat allele (*VNTR 3*) was more prevalent among HBV patients than HCs (OR = 1.4, 95% CI = 1.07–1.86, adjusted *P* = 0.017,). Genotype *VNTR 3/3* was observed more frequently in patients compared to HCs (OR = 1.4, 95% CI = 1.02–1.97, adjusted *P* = 0.036). Although the frequencies of *VNTRs 2/2* and *2/3* were higher among HCs than in the patient group, the differences were not significant.

**Table 2:** Association of the variable number of tandem repeats of *SMYD3* with HBV-related liver diseases.

*SMYD3* tandem repeat	HC n = 297 (%)	CHB n = 246 (%)	LC n = 174 (%)	HCC n = 336 (%)	CHB + LC n = 420 (%)	HBV total n = 756 (%)	CHB vs HC		LC vs HC		HCC vs HC		HBV vs HC	
							OR(95%CI)	*P* value	OR(95%CI)	*P* value	OR(95%CI)	*P* value	OR(95%CI)	*P* value
*Genotypes*														
*VNTR 2/2*	21 (7.1)	14 (5.7)	6 (3.4)	7 (2.1)	20 (4.8)	27 (3.6)	Reference		Reference		Reference		Reference	
*VNTR 2/3*	64 (21.5)	37 (15)	29 (16.7)	61 (18.2)	66 (15.7)	127 (16.8)	0.75 (0.32–1.76)	0.5	1.8 (0.59–5.6)	0.3	2.7 (0.89–7.92)	0.079	1.2 (0.58–2.44)	0.6
*VNTR 3/3*	212 (71.4)	195 (79.3)	139 (79.9)	268 (79.8)	334 (79.5)	602 (79.6)	1.2 (0.6–2.6)	0.5	2.2 (0.81–6.12)	0.1	**2.9(1.07–8)**	**0.037**	1.7 (0.88–3.15)	0.1
*Allele*														
*VNTR 2*	106 (21.7)	65 (13.2)	41 (11.8)	75 (11.2)	106 (12.6)	181 (12)	Reference		Reference		Reference		Reference	
*VNTR 3*	488 (78.3)	427 (86.8)	307 (88.2)	597 (88.8)	734 (87.4)	1331 (88)	1.4 (0.97–1.92)	0.078	1.5 (1–2.38)	0.05	**1.5 (1–2.14)**	**0.05**	1.4 (1.07–1.86)	**0.017**
*Dominant*														
*VNTR 2/2&2/3*	85 (28.6)	51 (20.7)	35 (20.1)	68 (20.2)	86 (20.5)	154 (20.4)	Reference		Reference		Reference		Reference	
*VNTR 3/3*	212 (71.4)	195 (79.3	139 (79.9)	268 979.8)	334 (79.5)	602 (79.6)	1.5 (0.99–2.23)	0.057	1.4 (0.88–2.4)	0.1	1.3 (0.86–2.05)	0.2	1.4 (1.02–1.97)	**0.036**
*Recessive*														
*VNTR 2/2*	21 (7.1)	14 (5.7)	6 (3.4)	7 (2.1)	20 (4.8)	27 (3.6)	Reference		Reference		Reference		Reference	
*VNTR 2/3&3/3*	276 (92.9)	232 (94.3)	168 (96.6)	329 (97.9)	400 (95.2)	729 (96.4	1.1 (0.55–2.35)	0.7	2.2 (0.8–6.02)	0.1	2.9 (1.07–8.03)	**0.036**	1.6 (0.82–2.94)	0.2

CHB, chronic hepatitis B; LC, liver cirrhosis; HCC, hepatocellular carcinoma; HC, healthy controls; n, numbers individuals; OR, Odd ratio. P values were calculated using binary logistic regression model adjusted for age and gender. Bold values reflect statistical significance.

We compared the distribution of allele and genotype frequencies of the *SMYD3* VNTRs in the patient subgroups (CHB, LC, HCC) with those in HCs. Genotype *VNTR 3/3* occurred more frequently in HCC patients than in HCs (OR = 2.9, 95%CI = 1.09–8, adjusted *P* = 0.037,), while genotype *VNTR 2/2* was nearly three times more frequent in HCs compared to HCC patients (HCC vs HC, OR = 0.34, 95% CI = 0.13–0.93, adjusted *P* = 0.036). However, no differences were observed in pairwise comparisons between the CHB, LC and HC groups.

We also analyzed associations of *SMYD3* VNTR polymorphisms with laboratory parameters of HBV infection (Alpha-fetoprotein (AFP), aspartate transaminase (AST), alanine aminotransferase (ALT), total and direct bilirubin, prothrombin, albumin, viral loads). The levels of these parameters were not different in all patients with different *SMYD3* VNTR genotypes as well as in patient subgroups (CHB, LC and HCC) (*P* > 0.05).

### SMYD3 serum levels in HBV-related liver diseases

**Figure 1:**
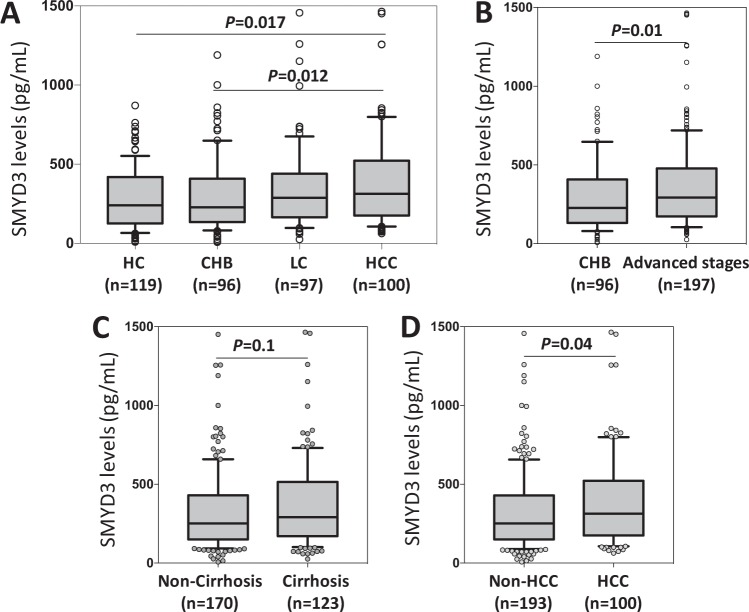
Association of the SMYD3 serum levels with HBV related liver diseases. The comparison of SMYD3 levels (**A**) among healthy controls and groups of patients according to HBV-related diseases, CHB, chronic hepatitis B; LC, liver cirrhosis without HCC; HCC, hepatocellular carcinoma; (**B**) between CHB patients with advanced stages patients (including LC and HCC), (**C**) between patients with and without cirrhosis, (**D**) between patients with and without HCC. Box-plots illustrate medians with 25 and 75 percentiles with whiskers to 10 and 90 percentiles; *P* value were calculated by using Kruskal-Wallis test or Mann-Whitney-Wilcoxon test.

Levels of SMYD3 increased significantly according to the progression of liver diseases (Fig. 1A,B). The SMYD3 median was 227.1 pg/mL in CHB patients, 287.3 pg/mL in LC patients and 311.8 in HCC patients and 292.6 pg/mL in patients with both HCC plus LC (*P* < 0.05). The post-hoc analysis showed a significant difference between the HCC and CHB groups (*P* = 0.012). A corresponding result was observed when comparing the median of SMYD3 levels in non-HCC and in HCC patients (251.1 pg/mL and 311.8 pg/mL, respectively, *P* = 0.04; Fig. 1D), whereas the median of SMYD3 levels did not differ between non-LC and LC individuals (Fig. 1C). These results indicate that SMYD3 plays a role in HCC development. When analyzing the correlation of SMYD3 serum levels with the laboratory parameters in all HBV patients and in patient subgroups, SMYD3 serum levels did not correlate with these parameters in all HBV patients and in the subgroups.

### *SMYD3* VNTRs and SMYD3 serum levels and progression of HBV-related liver diseases

**Figure 2:**
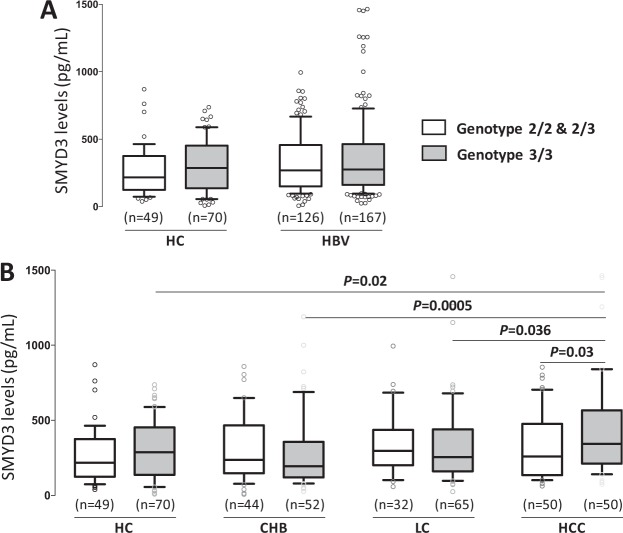
Association of the VNTRs of *SMYD3* with SMYD3 serum levels in HBV-related liver diseases. Comparison of the SMYD3 serum levels among healthy controls and patients with HBV-related liver diseases who carry different *SMYD3* VNTR genotypes. CHB, chronic hepatitis B, LC, liver cirrhosis without HCC, HCC, hepatocellular carcinoma. (**A**) Among healthy controls and HBV patients, (**B**) among healthy controls and patient subgroups including CHB, LC and HCC patients, Box-plots illustrate medians with 25 and 75 percentiles with whiskers to 10 and 90 percentiles; *P* values were calculated by using Mann-Whitney-Wilcoxon test or Kruskal-Wallis test.

We analyzed the relationship between *SMYD3* VNTR genotypes and SMYD3 serum levels in HBV patients (Fig. 2). SMYD3 levels in patients with *SMYD3* genotype 3/3 were similar to that in patients with genotypes 2/2 or 2/3 (*P* > 0.05, Fig. 2A). In the HCC group, patients with genotype *VNTR 3/3* had higher SMYD3 levels than those with genotypes *VNTR 2/2* and *2/3* (*P* = 0.03, Fig. 2B). This was not observed in CHB and LC patients (Fig. 2B). In addition, in patients with genotypes *2/2* and *2/3* the levels of SYMD3 did not differ between the CHB, LC and HCC groups. However, patients with genotype *VNTR 3/3* had increased SMYD3 serum levels according to the progression of liver diseases (HCC vs. CHB: *P* = 0.0005, LC vs. CHB: *P* = 0.036, Fig. 2B). These results indicate that genotype *VNTR 3/3* may increase SMYD3 levels and, thus, increase the risk of HCC development.

### *SMYD3* mRNA expression in HCC

**Figure 3:**
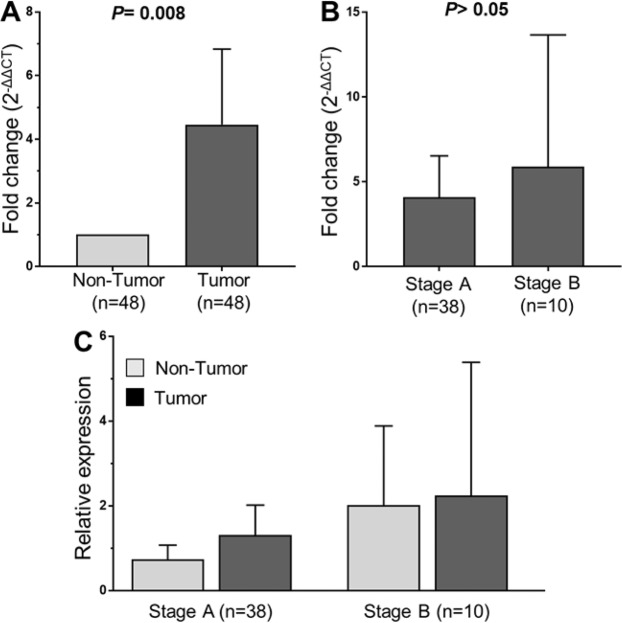
*SMYD3* mRNA expression in liver specimens from 48 HCC patients. Relative quantitative real-time PCR analysis of *SMYD3* mRNA levels. (**A**) Relative *SMYD3* mRNA expression in tumor tissues and in adjacent non-tumor tissues. (**B**) The fold changed *SMYD3* mRNA levels between the group of stage A-HCC patients and the group of stage B-HCC patients, (**C**) *SMYD3* mRNA expression in stage-A and stage-B tumor tissues and in adjacent non-tumor tissues. *P* values were calculated by using Mann-Whitney-Wilcoxon test. Data are shown as mean values with 95% confidence intervals.

In order to examine whether *SMYD3* mRNA is upregulated in cancer stages as seen in other cancers^14,15^, we analyzed the expression of *SMYD3* mRNA in HCC tissue specimens and in adjacent non-tumor liver tissues obtained from 48 HBV-related HCC patients. Expression of *SMYD3* mRNA in tumor tissues was significantly higher than that in adjacent non-tumor tissues (*P* = 0.008, Fig. 3A). We then examined whether *SMYD3* mRNA expression was associated with development of HCC by correlating *SMYD3* mRNA expression with BCLC stages. *SMYD3* mRNA expression was higher in stage-B tumor tissues compared to that in stage-A tissues (Fig. 3B). A similar trend was seen when *SMYD3* mRNA expression was compared between non-tumor tissues obtained from stage-A and stage-B HCC patients; however, the difference did not reach significance (Fig. 3C). These results indicate that SMYD3 expression is associated with HBV-related HCC.

## Discussion

HBV infection is a major cause of HCC. In Vietnam, the prevalence of HBV infection is over 10% in the general population^1,5^. Further to HBV, other factors contributing to the development of HCC include epigenetic changes such as methylation and histone modifications of regulatory genes^7,8,25^. SMYD3, a histone H3-K4 specific methyltransferase, is an example of histone modification which is considered a crucial epigenetic factor contributing to the development of various human cancers, including liver cancer^12,15^. So far, the role of SYMD3 and its encoding gene *SYMD3* in HBV-related liver diseases is not clear. We studied the association of genetic variation and expression of variant *SYMD3* VNTRs with susceptibility to HBV infection and with liver disease progression. We show that both *SMYD3* VNTRs in the promoter region and *SYMD3* overexpression are associated with HBV-related HCC.

*SMYD3* VNTR variability has been reported to be a significant factor in HCC^20,24^. An earlier study has reported that *VNTR 3/3* homozygosity conferred an over 3-fold increased risk of HCC in a Japanese population^20^. Accordingly, a study in a Chinese population found that the frequency of the *SMYD3* VNTR 3/3 genotype was higher in HCC patients than in controls^24^. Consistent with these studies, we observed that the frequencies of the *SMYD3* VNTR three repeat allele and the *3/3* genotype was higher in HBV patients than in controls (Table 2). Further analysis showed that in the HCC group the *SMYD3* repeat 3/3 genotype was more frequent than in HCs (OR = 2.9, *P* < 0.05, Table 2). Notably, Wang *et al*.^29^ did not find an association between the risk of HCC and the three tandem repeat allele.

The *SMYD3* VNTRs have been shown to be a susceptibility factor for human cancers, especially for colorectal cancer, breast cancer and HCC^20^. We observed that the *SMYD3* *VNTR 3/3* genotype increases the risk of HBV-related HCC. A possible explanation is that *SMYD3* *VNTR 3/3* can effectively promote its affinity with E2F-1, which is considered an important transcription factor stimulating cellular proliferation and cell cycle progression^20,30,31^. In HCC development, overexpression of SMYD3 was previously found in distinct cell lines^17,32^, and in HCC tumors^33^. However, an association between SMYD3 serum levels and HCC has not been shown so far. In our study, SMYD3 serum levels were significantly increased according to the various HBV-related liver disease stages. SMYD3 levels were higher in advanced liver disease (LC, HCC) compared to CHB, and HCC patients had higher SMYD3 levels than non-HCC patients. These findings indicate that increased SMYD3 levels may be associated with the occurrence of HCC, and SMYD3 serum levels may be considered a potential marker for HCC. Moreover, SMYD3 serum levels in HCC patients with the *SMYD3* VNTR genotype 3/3 were higher than in those with genotypes 2/2 and 2/3; this difference was not observed in CHB and LC patients.

Finally, we assessed *SMYD3* mRNA expression in liver tissues. *In vitro* evidence indicates that SMYD3 expression is up-regulated in HCC cell lines^12,32^. SMYD3 expression was analyzed in HCC tissues, and upregulation was significantly associated with an unfavourable prognosis of HCC^33,34^. *SMYD3* mRNA expression was upregulated in HCC tumor compared to adjacent non-tumor tissues, providing further evidence of a role of SMYD3 in HCC development. Several studies have shown that SMYD3 can interact with the HBV-HBx protein, which can induce upregulation of SMYD3 in HCC, these interactions promote the development of HCC by regulating ERK- and AKT/GSK-3β signaling pathways^17–19,35^. The *SMYD3* VNTR polymorphisms might regulate HCC development possibly by increased *SMYD3* expression in the liver. A recent study has shown that SMYD3 may interact with cyclin-dependent kinase 2 (CDK2) and matrix metallopeptidase 2 (MMP2), which are involved in controlling cell cycle, proliferation and invasion of hepatocytes, and thus contributing to the tumorigenicity of HCC^33^. SMYD3 was also associated with ankyrin repeat and KH domain-containing protein 1 (ANKHD1) that regulate the initiation, progression and metastasis in HCC^36^ Although our data underline the significance of the *SMYD3* VNTR 3/3 genotype and SMYD3 overexpression in HBV-related HCC development, the interaction between HBV and SYMD3 needs to be investigated further.

In conclusion, our study shows the association of the *SMYD3* *VNTR 3/3* as well as upregulation of SMYD3 serum levels and mRNA expression with HCC development, suggesting that SYMD3 has some potential for the prediction of HCC in HBV patients.
